# A multifaceted clinical decision support intervention to improve adherence to thromboprophylaxis guidelines

**DOI:** 10.1007/s11096-021-01254-x

**Published:** 2021-03-11

**Authors:** Tessa Jaspers, Marjolijn Duisenberg-van Essenberg, Barbara Maat, Marc Durian, Roy van den Berg, Patricia van den Bemt

**Affiliations:** 1 Department of Hospital Pharmacy, Elisabeth TweeSteden Hospital, Dr. Deelenlaan 5, 5042 AD Tilburg, The Netherlands; 2 Department of Oncology and Hematology, Elisabeth TweeSteden Hospital, Hilvarenbeekse Weg 60, 5022 GC Tilburg, The Netherlands; 3 Intensive Care Unit, Elisabeth TweeSteden Hospital, Hilvarenbeekse Weg 60, 5022 GC Tilburg, The Netherlands; 4 Department of Clinical Pharmacy and Pharmacology, University Medical Center Groningen, Hanzeplein 1, 9713 GZ Groningen, The Netherlands

**Keywords:** Clinical, Decision support systems, Electronic health records, Guideline Adherence, Heparin, Low-molecular-weight, Venous Thromboembolism

## Abstract

*Background* Venous thromboembolism is a potentially fatal complication of hospitalisation, affecting approximately 3% of non-surgical patients. Administration of low molecular weight heparins to the appropriate patients adequately decreases venous thromboembolism incidence, but guideline adherence is notoriously low. *Objective* To determine the effect of a multifaceted intervention on thromboprophylaxis guideline adherence. The secondary objective was to study the effect on guideline adherence specifically in patients with a high venous thromboembolism risk. As an exploratory objective, we determined how many venous thromboembolisms may be prevented. *Setting* A Dutch general teaching hospital. *Method* A prospective study with a pre- and post-intervention measurement was conducted. A multifaceted intervention, consisting of Clinical Decision Support software, a mobile phone application, monitoring of duplicate anticoagulants and training, was implemented. Guideline adherence was assessed by calculating the Padua prediction and Improve bleeding score for each patient. The number of preventable venous thromboembolisms was calculated using the incidences of venous thromboembolism in patients with and without adequate thromboprophylaxis and extrapolated to the annual number of admitted patients. *Main outcome measure* Adherence to thromboprophylaxis guidelines in pre- and post-intervention measurements. *Results* 170 patients were included: 85 in both control and intervention group. The intervention significantly increased guideline adherence from 49.4 to 82.4% (OR 4.78; 95%CI 2.37–9.63). Guideline adherence in the patient group with a high venous thromboembolism risk also increased significantly from 54.5 to 84.3% (OR 2.46; 95%CI 1.31–4.62), resulting in the potential prevention of ± 261 venous thromboembolisms per year. *Conclusions* Our multifaceted intervention significantly increased thromboprophylaxis guideline adherence.

## Impacts on practice

- Our multifaceted intervention significantly increases overall guideline adherence from 49.4 to 82.4%.
- Implementing this multifaceted intervention globally may prevent almost half of all venous thromboembolisms in non-surgical patients.
- We advocate the use of highly specific and user-friendly clinical decision support to address similar problems in healthcare.

## Introduction

Approximately 3% of in-hospital non-surgical patients develop a venous thromboembolism (VTE) without adequate prophylaxis [1]. This rises to 11% in patients that have a higher VTE risk. VTEs are complications with a high morbidity and mortality. Around 40% of VTEs are pulmonary embolisms, which is one of the most common causes of death in hospitals worldwide [2–4]. Correct usage of thromboprophylaxis can reduce the incidence of VTEs in high risk patients by approximately 80% [1]. On the other hand, thromboprophylaxis increases bleeding risk, with a potential morbidity and mortality as well. Adequate use of thromboprophylaxis is therefore an important factor in patient safety and quality of healthcare.

Advice on when to prescribe thromboprophylaxis has been included in various international guidelines, such as The American College of Chest Physicians (ACCP) guideline thrombosis prophylaxis 2012 and the Dutch Internists Association (NIV) guideline anti-thrombotic policy 2015 [5, 6]. Both guidelines are similar as to when to start prophylaxis as they both use the same risk assessment models (RAMs): the Padua prediction score for VTE risk and the Improve bleeding matrix for bleeding risk [1, 5–7].

According to these guidelines, thromboprophylaxis, preferably in the form of low molecular weight heparins (LMWHs), should be started if a patient has a high risk of VTE. If the patient also has a bleeding risk, mechanical prophylaxis in the form of elastic stockings or intermittent pneumatic compression should be started [5, 6].

However, low adherence to thromboprophylaxis guidelines is described in literature. An international cross-sectional study from 32 countries stated that, on average, 39.5% of non-surgical patients with a high VTE risk received adequate prophylaxis [8]. The Netherlands Institute for Research of Healthcare (NIVEL) reported that in 13 Dutch hospitals an average of 60% of non-surgical patients with a high VTE risk received thromboprophylaxis [9]. In addition; 37% of patients with an increased bleeding risk, without a high VTE risk, received thromboprophylaxis. In the patient group with an increased bleeding risk and a high VTE risk, 60% of patients received chemical thromboprophylaxis instead of mechanical prophylaxis.

The Cochrane Collaboration published two systematic reviews about several methods that may increase guideline adherence of thromboprophylaxis in non-surgical patients [10, 11]. Single interventions with training, posters and/or pocket cards demonstrated as ineffective in improving guideline adherence [10, 12–15]. Multifaceted interventions resulted in a mean improvement of only 4% [11, 16, 17]. Randomized controlled trials with computer based alerts demonstrated an improvement of 0–16% in guideline adherence, with the use of non-specific alerts [11, 18, 19]. Results from single-arm studies with Clinical Decision Support (CDS) also demonstrated substantial heterogeneity, with improvements rates of 0–23% [20–23]. The CDS used in these studies were also non-specific, such as continuous flashing reminders when thromboprophylaxis had not been started, posing a high risk of alert fatigue [18, 20–23].

To our knowledge, no study has described the effect of a multifaceted intervention with a highly specific CDS. Therefore, we aimed to implement a multifaceted intervention with a user-friendly CDS and highly specific design. To achieve this level of specificity, the Padua Prediction Score was built into the electronic health record (EHR), making it possible to alert only if relevant.

## Aim of the study

The aim of this study was to determine the effect of a multifaceted intervention, consisting of a highly specific CDS, training, a mobile phone application and monitoring on duplicate anticoagulant medication by a pharmacist, on guideline adherence in non-surgical patients. The secondary objective was to study the effect of the multifaceted intervention on guideline adherence in patients with a high VTE risk (≥ 4 points on the Padua prediction score). Exploratory objective was to calculate how many VTEs may be prevented annually in our hospital by implementing the multifaceted intervention.

## Methods

### Study design and setting

This study was a prospective intervention study with a pre- and post-intervention measurement. It was conducted at the Elisabeth TweeSteden Hospital (ETZ), a large general teaching hospital and traumacenter with 996 beds in Tilburg, The Netherlands.

### Study population

Non-surgical patients, ≥ 18 years of age, admitted to the departments of neurology, internal medicine or oncology & haematology, with a hospital stay of ≥ 36 h in the pre- or post-intervention period have been included. Patients with orders for comfort measures only have been excluded.

### Multifaceted intervention

Before the introduction of the multifaceted intervention, the initiation of thromboprophylaxis was left to the discretion of the physician, who could consult locally available web based protocols and (inter)national guidelines on thromboprophylaxis.

The multifaceted intervention was implemented from November 2018 through February 2020 and consisted of the following components: the introduction of a mobile phone application ‘Pocket Cards’ (Interactive Studios, Rosmalen, The Netherlands), a clinical rule ‘duplicate anticoagulant medication’, CDS in the form of a Best Practice Advisory (BPA) in the Electronic Health Record (EHR, Epic^®^, Epic Systems, Verona, USA) and training of prescribers as described in Table 1.

**Table 1:** Overview of the intervention components [1, 32]

When	Component	Description
November 2018	Mobile phone application ‘Pocket Cards’	A decision support mobile phone application, based on the Padua prediction score, could be consulted by the prescriber at any time to decide whether to start thromboprophylaxis. Risk factors of a patient must be entered manually in this application, with no link to the EHR
July 2019	Clinical rule ‘duplicate anticoagulant medication’	A patient list in the EHR, automatically selecting patients with combinations of thromboprophylaxis (ATC code B01AB) and therapeutic anticoagulation (ATC codes B01AA, B01AE and B01AF), was assessed daily by a pharmacist for rationale of combinations of anticoagulants. In the event of an incorrect combination, the pharmacist advised the prescriber to discontinue thromboprophylaxis
December 2019	Training	Training of prescribers on the wards neurology, internal medicine and oncology and hematology, covering the incidence of VTEs in non-surgical patients, the effect of thromboprophylaxis on the incidence of VTEs and the results of the control group data collection. A demonstration of CDS, which would be implemented in February 2020, was given
February 2020	CDS	An advanced CDS, aggregating data from the EHR, gave an automated advice to the physician in the EHR whether thromboprophylaxis was necessary according to the Padua prediction score. To this end, the CDS collects data from the patients’ problem list (e.g. malignancy, VTE in the past, thrombophilia), patient characteristics (sex, age, weight, BMI), the medication list (hormonal treatment and anticoagulants) and from the mobility score of the Braden score (mobility), which is assessed for each patient in our hospital within 24 h after admission [1, 32]. If the Padua score is ≥ 4 and no anticoagulant is in use, the CDS generates an advice (pop-up) to the prescriber to initiate medicinal thromboproophylaxis. This advice is adapted to the weight and BMI of the patient; an order for dalteparin 2500 IE is suggested in patients < 90 kg and BMI < 30 kg/m^2^, otherwise dalteparin 5000 IE is suggested. Because the bleeding risk is not included in the CDS, a general disclaimer is included that the prescriber must consider the bleeding risk

*EHR* Electronic health record, *ATC* anatomical therapeutic chemical, *VTE* venous thromboembolism, *CDS* clinical decision support

### Outcome measures

The primary outcome was the proportion of patients for whom thromboprophylaxis was prescribed according to guidelines. The secondary outcome was to determine the effect of the intervention on guideline adherence specifically in patients with a high VTE risk.

Guideline adherence was assessed using the Padua Prediction Score and Improve bleeding RAM, as included in Table 2 [1, 7]. The VTE risk was considered high at a Padua Prediction score of  ≥ 4 [1]. The bleeding risk was considered high at an Improve score of  ≥ 7, or when a patient scored positive on the high-risk factors: prior bleeding in the last 3 months, an active gastro-duodenal ulcer or a platelet count < 50 × 10^9^/L [7]. According to (inter)national guidelines, chemical thromboprophylaxis should be initiated if a patient has a high risk of VTE. If the patient also has an increased bleeding risk, mechanical prophylaxis in the form of elastic stockings or intermittent pneumatic compression should be started [5, 6].

**Table 2:** Padua prediction score and improve bleeding risk assessment tool [1, 7]

Padua prediction score		Improve bleeding risk	
High risk of VTE: ≥ 4		High risk of bleeding: ≥ 7, or ≥ 1 of the high-risk factors prior bleeding (< 3 months), active gastric or duodenal ulcer or platelet count less than 50 × 10^9^/L	
Risk factor	Score	Risk factor	Score
Active cancer^a^	3	Moderate renal failure (eGFR 30–50 ml/min)	1
Previous VTE^b^	3	Male sex	1
Reduced mobility^c^	3	40–84 years	1.5
Thrombophilic condition^d^	3	Active cancer	2
Recent (≤ 1 month) trama and/or surgery	2	Rheumatic disease	2
Age (≥ 70 years)	1	Central venous catheter	2
Heart and/or respiratory failure	1	Admission in Intensive Care Unit	2.5
Acute MI or ischemic stroke	1	Sever renal failure (< 30 ml/min)	2.5
Acute infection and/or rheumatologic disorder	1	Liver insufficiency (INR > 1.5)	2.5
BMI ≥ 30 kg/m^2^	1	≥ 85 years	3.5
Hormonal treatment	1	Thrombocytopenia (< 50 × 10^9^ cell/L)	4
		Recent (< 3 months) bleeding	4
		Active gastro-intestinal ulcer	4.5

*VTE* Venous thromboembolism, *MI* myocardial infarction, *BMI* body mass index, *eGFR* estimated glomerular filtration rate, *INR* international normalized ratio

^a^Patients with local or distant metastases and/or in whom chemotherapy or radiotherapy had been performed in the previous 6 months

^b^Superficial vein thrombosis excluded

^c^Bedrest with bathroom privileges [either due to patient’s limitations or on physicians order] for at least 3 days

^d^Carriage of defects of antithrombin, protein C or S, factor V Leiden, G20210A prothrombin mutation, antiphospholipid syndrome

The exploratory outcome was to calculate how many VTEs may theoretically be prevented annually in the ETZ by the multifaceted intervention. Therefore, the annual number of patients admitted to the ETZ with a high VTE risk and no bleeding risk was calculated. To this end, an annual admission rate of 25,000 patients and a prevalence of 39.7% patients with a high VTE risk without bleeding risk was used, resulting in 9,925 patients [1]. Subsequently, the expected number of VTEs in the pre-intervention (T0) and post-intervention (T1) period was calculated based on the percentages of correctly and incorrectly treated patients with a high VTE risk. A VTE incidence rate of 2.2% was used in the patient group with thromboprophylaxis and 11.0% in the patient group without thromboprophylaxis. Finally the expected number of preventable VTEs was calculated as the difference between T0 and T1 [1].

### Data collection

Assessment of guideline adherence was done once for each patient between ≥ 36 and ≤ 60 h after admission by one pharmacist (TJ). When in doubt, a hematologist (MD) was consulted. Pre-intervention data collection was performed retrospectively over one month (T0, October 2018). Post-intervention data collection was performed prospectively over one month (T1, March 2020).

In both periods, a patient list was generated from the EHR with all patients who met the inclusion criteria. For each patient, risk factors included in the Padua Prediction Score and Improve bleeding RAM were collected from the patient’s EHR.

Patient characteristics and drug use were mostly discrete data in the EHR (e.g. values from pre-populated lists, coded data or data entered into fields requiring specific alphanumeric formats). However, it is possible that not all risk factors for VTE and bleeding were documented as discrete data. To collect the non-discrete data, every risk factor was searched for manually in the free text notes in the patient’s EHR.

### Data monitoring

Patient data were coded and processed in Datamanager 5.43.0 (The research manager, Deventer, The Netherlands).

### Data analysis

The sample size was calculated using a p-value of 0.05, 80% power, a 60% rate of guideline adherence prior to the multifaceted intervention, and an expected increase of 20% in adequacy following the multifaceted intervention [8–10]. A Chi-square test calculation resulted in 81 patients per group. In order to ensure the required sample size, we increased the number of patients to 85 per group, thus 170 patients in total.

Data analysis was performed with IBM SPSS Statistics vs 24 (IBM, New York, USA). Categorical variables were presented as proportions (in %) and continuous variables were presented as mean (with standard deviation) if the data were normally distributed or with a median (with an interquartile range) if the data were not normally distributed. Differences in patient characteristics between groups were tested using Pearson X^2^ test for categorical variables, or Fisher’s exact test when the expected number of cases per cell was ≤ 5. Unpaired t-tests were used for continuous variables. The Mann–Whitney U test was used when variables were not parametric.

The difference in the percentage of correctly treated patients between T0 and T1 was analyzed by univariate logistic regression. Common confounding variables such as age and sex and the highest scoring VTE risk factors in the Padua prediction score (immobility, malignancy and VTE in the past) were included in the multivariate logistic regression [1, 24]. Moreover, length of stay was chosen as a confounding variable as this may influence the initiation of thromboprophylaxis. The admission department was chosen as this varied the most (p = 0.17) between the two groups. Covariates were chosen according to the Stepwise regression with backward elimination, with a cut-off p-value < 0.2 for covariate selection. Covariates immobility, malignancy and VTE in the past were included in the final model.

Results were presented as odds ratios (OR) with 95% confidence intervals (95%CI). All statistics were 2-tailed, and p < 0.05 was considered to be statistically significant.

## Results

172 patients were reviewed, 85 in T0 and 87 in T1. None of the patients in T0 were excluded, while two patients were excluded in T1 because of a comfort measures only policy. In total, 170 patients were included; 85 in both T0 and T1.

Table 3 summarizes the patient characteristics. There are no significant differences between the two patient groups. In both groups, the mean age was 66 years and men represented a small majority. The median length of stay was 8.3 days in T0 and 7.4 days in T1. A high VTE risk was present in 76.5 and 70.6% in T0 and T1 (p = 0.39), respectively. A high bleeding risk was present in 15.3 and 14.1% in T0 and T1 (p = 0.83), respectively.

**Table 3:** Patient characteristics

Variable	T0 [n = 85]	T1 [n = 85]	P-value
Patient characteristics			
Male sex, n [%]	49 [57.6]	52 [61.2]	0.64
Age, mean years ± SD	65.8 ± 16.9	66.0 ± 16.8	0.95
Length of stay, median days ± SD	8.3 ± 8.6	7.4 ± 5.1	0.41
Weight, mean kg ± SD	77.8 ± 17.4	76.6 ± 17.6	0.67
BMI, mean kg/m^2^ ± SD	26.1 ± 5.0	26.2 ± 5.5	0.92
Therapeutic anticoagulation in use, n [%]	16 [18.8]	22 [25.9]	0.27
High risk of VTE^a^, n [%]	65 [76.5]	60 [70.6]	0.39
High risk of bleeding^b^, n [%]	13 [15.3]	12 [14.1]	0.83
High VTE and bleeding risk, n [%]	10 [11.8]	9 [10.6]	0.81
High VTE risk, without risk of bleeding, n [%]	55 [64.7]	51 [60.0]	0.53
Department, n [%]			0.17
Internal Medicine	34 [40.0]	24 [28.2]	–
Neurology	29 [34.1]	29 [34.1]	–
Oncology and haematology	22 [25.9]	32 [37.6]	–
Risk factors for VTE, n [%]			
Active cancer	26 [30.6]	28 [32.9]	0.74
Previous VTE	15 [17.6]	12 [14.1]	0.53
Reduced mobility	55 [64.7]	50 [58.8]	0.43
Trombophilic condition	0 [0.0]	1 [1.2]	1.00
Recent (≤ 1 month) trauma and/or surgery	7 [8.2]	7 [8.2]	1.00
Age (≥ 70 years)	41 [48.2]	44 [51.8]	0.65
Heart and/or respiratory failure	27 [31.8]	28 [32.9]	0.87
Acute MI or ischemic stroke	14 [16.5]	17 [20.0]	0.55
Acute infection	30 [35.3]	33 [38.8]	0.63
Rheumatic disease	28 [32.9]	24 [28.2]	0.51
BMI ≥ 30 kg/m2	17 [20.0]	17 [20.0]	1.00
Hormonal treatment	2 [2.4]	0 [0.0]	0.49
Padua score, mean ± SD	5.4 ± 2.8	5.3 ± 3.1	0.86
Risk factors for bleeding, n [%]			
Age category			0.57
0—39 years	8 [9.4]	7 [8.2]	–
40–84 years	66 [77.6]	71 [83.5]	–
≥ 85 years	11 [12.9]	7 [8.2]	–
Renal failure (eGFR < 50 ml/min)	17 [20.0]	19 [22.4]	0.71
Active cancer	26 [30.6]	28 [32.9]	0.74
Rheumatic disease	28 [32.9]	24 [28.2]	0.51
Central Venous catheter	9 [10.6]	7 [8.2]	0.60
Hepatic failure (INR > 1,5)	2 [2.4]	3 [3.5]	1.00
Platelet count < 50 × 10^9^ cells/l	1 [1.2]	3 [3.5]	0.62
Recent bleeding (≤ 3 months)	7 [8.2]	8 [9.4]	0.79
Active gastroduodenal ulcer	2 [2.4]	2 [2.4]	1.00
Improve bleed score, mean ± SD	4.3 ± 2.4	4.3 ± 2.5	0.82

*T0* Pre-intervention measurement, *T1* post-intervention measurement, *SD* standard deviation, *VTE* venous thromboembolism, *MI* myocardial infarction, *BMI* body mass index, *eGFR* estimated glomerular filtration rate; *INR* international normalized ratio

^a^Padua score ≥ 4

^b^Improve score ≥ 7, or ≥ 1 of the high-risk factors prior bleeding in the last 3 months, active gastric or duodenal ulcer or platelet count less than 50 × 10^9^/L

### Overall guideline adherence

The percentage of patients in whom thromboprophylaxis was prescribed adherent to guidelines is shown in Table 4. The adherence was 49.4% in T0 and 82.4% in T1 (OR 4.78; 95%CI 2.37–9.63) and increases after adjustment for immobility, malignancy and VTE in the past (OR_adj_ 5.88; 95%CI 2.74–12.62). Both a significant increase in patients who correctly received thromboprophylaxis and in patients who correctly did not receive thromboprophylaxis was observed.

**Table 4:** Adherence to thromboprophylaxis guidelines before (T0) and after (T1) intervention

Classification of treatment	T0 [n = 85]	T1 [n = 85]	OR [95%CI]	Adjusted OR [95%CI]^a^
Overall guideline adherence, n [%]	42 [49.4]	70 [82.4]	4.78 [2.37–9.63]*	[2.74–12.62]*
Thromboprophylaxis according to guidelines	17 [20.0]	30 [35.3]	2.18 [1.09–4.36]*	[1.21–5.42]*
No thromboprophylaxis according to guidelines	25 [29.4]	40 [47.1]	2.13 [1.13–4.01]*	2.59 [1.21–5.58]*

*T0* Pre-intervention measurement, *T1* post-intervention measurement, *OR* odds ratio, *CI* confidence interval

*Statistically significant (95%CI > 1.00)

^a^Adjusted for immobility, malignancy and VTE in the past

### Guideline adherence in high risk VTE patients

There were 55 patients with a high VTE risk, without a bleeding risk in T0. This is comparable to 51 patients in T1. Guideline adherence in this patient group increased significantly from 54.5 to 84.3% (OR 2.46; 95%CI 1.31–4.62) in T1 compared to T0. This effect was larger when adjusted for immobility, malignancy and VTE in the past (OR_adj_ 4.00; 95%CI 1.86–8.59).

### Number of VTEs that may be prevented

**Fig. 1:**
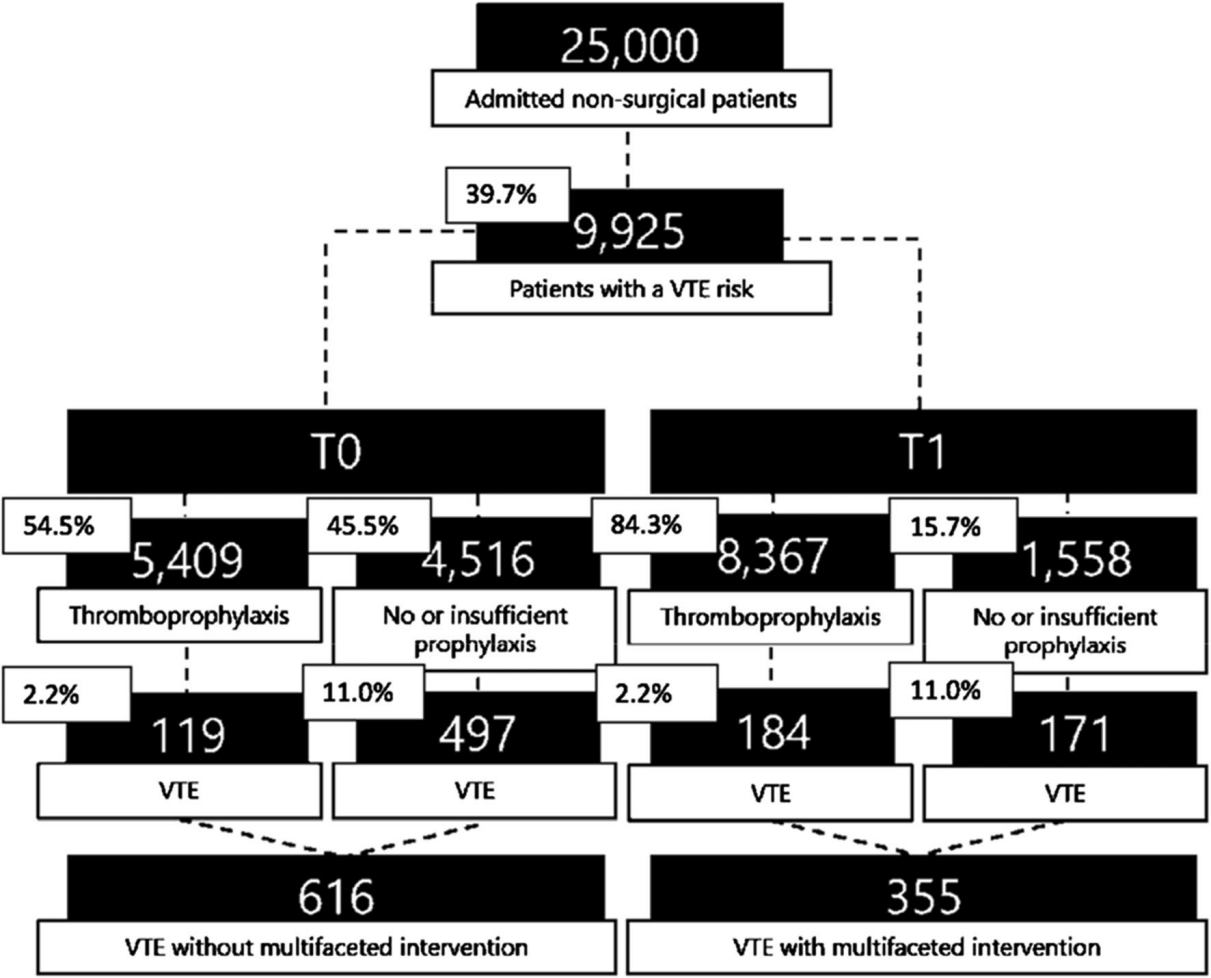
The number of venous thromboembolisms that may be prevented based on extrapolation to the annual number of admitted patients. *VTE *venous thromboembolism, *T0* pre-intervention measurement; *T1* post-intervention measurement. The percentage of adherence and non-adherence to guidelines in T0 and T1 is extrapolated to the total number of patients with a high VTE risk (9,925). Subsequently, a VTE incidence of 2.2% is considered in the population receiving adequate thromboprophylaxis, compared to 11.0% in the population receiving no or inadequate thromboprophylaxis

The number of venous thromboembolisms in T0 and T1 extrapolated to the annual number of admitted patients in the ETZ, is shown in Fig. 1. Considering an annual admission rate of 25,000 patients, 9925 (39.7%) patients will have a high VTE risk, without a bleeding risk [1]. In T0, guideline adherence was 54.5%; 5409 of 9925 patients with a high VTE risk would have received thromboprophylaxis adequately. Of these patients approximately 119 (2.2%) would develop a VTE. Non-guideline adherence was 45.5%; 4516 patients with a high VTE risk would not have been treated with thromboprophylaxis, of which 497 patients (11.0%) would develop a VTE [1]. In theory, a total of 616 VTEs would occur in our hospital annually, considering the guideline adherence in T0.

In T1, guideline adherence increased to 84.3%. In this case, 8367 of 9925 patients with a high VTE risk would receive adequate thromboprophylaxis. Approximately 184 (2.2%) of these patients would develop a VTE. Non-guideline adherence was 15.7%; 1558 patients with a high VTE risk would not have been treated with thromboprophylaxis, of which 171 (11.0%) would develop a VTE. In theory, a total of 355 VTEs would occur in our hospital annually considering the guideline adherence in T1.

In conclusion, approximately 616 VTEs would occur annually without the multifaceted intervention and 355 VTEs would occur annually with all interventions in place. Therefore, the intervention potentially prevents 261 of 616 (42.3%) VTEs every year.

## Discussion

The multifaceted intervention significantly increased overall guideline adherence from 49.4 to 82.4%, respectively. Also, in the patient group with a high VTE risk guideline adherence improved significantly from 54.5 to 84.3%. Extrapolation of these results to an annual admission rate of 25,000 patients in our hospital, results in a potential decrease of ± 261 (42.3%) VTEs per year.

To our knowledge, this is the first study describing such a large effect on guideline adherence after the implementation of a multifaceted intervention. We believe this is mostly due to the highly specific and user-friendly design of our CDS. For example, the CDS is integrated in the EHR to ensure hospital wide availability. Also, the CDS has a highly specific ‘focused’ design to prevent alert fatigue; prescribers are only alerted if patients actually have a high VTE risk and are not treated with anticoagulant therapy. To achieve this level of specificity, the CDS design and build were based on (derivatives of) discrete data, a time consuming but worthwhile process that led to the immediate availability of the Padua Prediction Scores in the EHR. Additionally, thromboprophylaxis could be prescribed with a single click in order to make it easy for prescribers ‘to do the right thing’.

Various studies have described that these characteristics are important factors for a successful CDS. For example, Eijgenraam et al. investigated the use of CDS, which was only available on a limited number of computers that were unable to communicate with the EHR [23]. Moreover, Kucher et al. investigated the effect of a multifaceted intervention, consisting of training for prescribers and a non-specific CDS in the form of continuously flashing non-interruptive alerts in the EHR when thromboprophylaxis was not ordered [18, 20] This intervention resulted in a moderate guideline adherence increase of 23%. To our knowledge, no other study described the effect of a multifaceted intervention, with a highly-specific CDS incorporating the Padua Prediction Score in the EHR.

It is interesting to know which intervention has the most impact. The CDS seems to have a large effect in the post-intervention group. In March 2020, the month in which the post-intervention data collection took place, 2,100 patients were admitted, the CDS alert was shown to prescribers 1144 times and was accepted 401 times (data not shown). This alone already leads to an increase of approximately 19% in compliance with guidelines.

In contrast to our expectations, the clinical rule ‘duplicate anticoagulant medication’ had little effect on the primary outcome measure. None of the enrolled patients in T1 had received an intervention based on the combination of thromboprophylaxis and therapeutic anticoagulation, while 16 (18.8%) and 22 (25.9%) patients were on therapeutic anticoagulation in T0 and T1, respectively. The effect of the phone application and training of prescribers is difficult to measure.

Our study has several limitations. Only non-surgical patients were included in this study, because the use of thromboprophylaxis is different between surgical and non-surgical patients. In our hospital, the perioperative anticoagulation policy is covered in electronic standardized ordersets readily available in the EHR and therefore would need a different approach to improve adherence [4]. Moreover, multiple studies have described that the administration of appropriate thromboprophylaxis in non-surgical patients is worse than in surgical patients [5, 25–27].

The period between T0 and T1 was long (14 months), potentially resulting in the influence of factors outside our multifaceted intervention. This period was necessary for the various interventions to be built and implemented. In this time period, there were no changes in our guidelines for thromboprophylaxis, EHR usage, training or prescription policy on this topic in our hospital. We believe that the influence of factors, other than the ones described above, is small due to the broad, comprehensive design of the interventions. We do not expect that the time difference may explain the increase in guideline adherence. As described in several articles, the guideline adherence in T0 is comparable with several international studies [8, 9, 18, 21–23].

The results of this study are dependent on the documentation in the EHR by practitioners in two ways: risk factors must be documented in the EHR, otherwise they could be missed in the data analysis of T0 and T1. In addition, risk factors such as active cancer, previous VTE and rheumatic disease must be documented in the so-called ‘problem list’ for the CDS to be able to include them. Otherwise, these risk factors are omitted in the calculated Padua Prediction Score. Numerous studies have described the incompleteness of patients’ problem lists in hospitals, causing a lower effectiveness of CDS [28, 29].

Future study should address whether other hospitals will show the same results with this multifaceted intervention and if the increase in guideline adherence is sustained over time. Moreover, several studies have reported a 90% or more guideline adherence after the implementation of dedicated multidisciplinary teams [30, 31]. Our multifaceted intervention, combined with the implementation of such a multidisciplinary team might further increase guideline adherence in a sustained matter and should be studied too [30, 31].

## Conclusion

A multifaceted intervention, including a highly specific CDS for high-risk VTE patients, has demonstrated to increase adherence to thromboprophylaxis guidelines significantly with 33%. This potentially prevents 261 VTEs per year in our hospital.
